# Mannan adjuvants intranasally administered inactivated influenza virus in mice rendering low doses inductive of strong serum IgG and IgA in the lung

**DOI:** 10.1186/s12879-015-0838-7

**Published:** 2015-02-26

**Authors:** Owen Proudfoot, Sandra Esparon, Choon-Kit Tang, Karen Laurie, Ian Barr, Geoffrey Pietersz

**Affiliations:** Bio-organic and Medicinal Chemistry Laboratory, Centre for Biomedical Research, Burnet Institute, 85 Commercial Road, Melbourne, 3004 Australia; Immunology Frontier Research Centre, 6F IFReC Research Building, 3-1 Yamada-oka, Suita, Osaka Japan; WHO Collaborating Centre for Reference and Research on Influenza, 10 Wreckyn Street North, Melbourne, 3051 Australia; Department of Pathology, University of Melbourne, Parkville, Victoria Australia; Department of Immunology, Monash University, Melbourne, Victoria Australia

**Keywords:** Adjuvant, Dose-sparing, H1N1, IgA, Influenza, Immunisation, Intranasal, Mannan, Mucosal immunity, Vaccine

## Abstract

**Background:**

H1N1 influenza viruses mutate rapidly, rendering vaccines developed in any given year relatively ineffective in subsequent years. Thus it is necessary to generate new vaccines every year, but this is time-consuming and resource-intensive. Should a highly virulent influenza strain capable of human-to-human transmission emerge, these factors will severely limit the number of people that can be effectively immunised against that strain in time to prevent a pandemic. An adjuvant and mode of administration capable of rendering ordinarily unprotective vaccine doses protective would thus be highly advantageous.

**Methods:**

The carbohydrate mannan was conjugated to whole inactivated H1N1 influenza virus at a range of ratios, and mixed with it at a range of ratios, and various doses of the resulting preparations were administered to mice via the intranasal (IN) route. Serum immunity was assessed via antigen-specific IgG ELISA and the haemagglutination-inhibition (HI) assay, and mucosal immunity was assessed via IgA ELISA of bronchio-alveolar lavages.

**Results:**

IN-administered inactivated H1N1 mixed with mannan induced higher serum IgG and respiratory-tract IgA than inactivated H1N1 conjugated to mannan, and HIN1 alone. Adjuvantation was mannan-dose-dependent, with 100 μg of mannan adjuvanting 1 μg of H1N1 more effectively than 10 or 50 μg of mannan. Serum samples from mice immunised with 1 μg H1N1 adjuvanted with 10 μg mannan did not inhibit agglutination of red blood cells (RBCs) at a dilution factor of 10 in the HI assay, but samples resulting from adjuvantation with 50 and 100 μg mannan inhibited agglutination at dilution factors of ≥ 40. Both serum IgG_1_ and IgG_2a_ were induced by IN mannan-adjuvanted H1N1 vaccination, suggesting the induction of humoral and cellular immunity.

**Conclusions:**

Mixing 100 μg of mannan with 1 μg of inactivated H1N1 adjuvanted the vaccine in mice, such that IN immunisation induced higher serum IgG and respiratory tract IgA than immunisation with virus alone. The serum from mice thus immunised inhibited H1N1-mediated RBC agglutination strongly *in vitro*. If mannan similarly adjuvants low doses of influenza vaccine in humans, it could potentially be used for vaccine ‘dose-sparing’ in the event that a vaccine shortage arises from an epidemic involving a highly virulent human-to-human transmissable influenza strain.

## Background

Currently, H1N1 vaccines are administered unadjuvanted intramuscularly and rely on the induction of serum IgG to induce protection. Estimating the vaccine efficacy (VE) of this strategy in a broad sense is complicated by the fact that from year to year the strains chosen for inclusion match the actual emerging strains to varying degrees; thus data collected one year cannot be directly compared to data collected the next. Vaccination is clearly far from 100% efficacious, with a recent large-scale 'sentinel' study calculating an overall VE of 47% [1]. Immunising with live attenuated virus at the site of influenza virus entry, *i.e.* intranasally (IN), has been investigated in humans and results suggest that a regime based on or including IN immunisation may enhance VE, and may be more effective in generating heterotypic immunity [2,3].

Recent threats of the potential large-scale emergence of human-to-human transmissible forms of virulent H5N1 (reviewed in Kaplan *et al*. [4]) and H7N9 (reviewed in Wu *et al*. [5]) avian influenza strains, and the actual emergence of widespread human-to-human transmissible variants of an H1N1 variant dubbed ‘swine-flu’ (reviewed in Dhama *et al*. [6]) have highlighted the need for a non-toxic, cost-effective dose-sparing influenza vaccine adjuvant. An adjuvant that substantially reduces the amount of influenza virus normally required for protective vaccination would allow for faster immunisation of potentially high-risk sections of the population such as children and asthmatics, against a rapidly emerging, highly virulent influenza strain.

In this study, we investigated the ability of mannan to adjuvant whole inactivated H1N1 (A/New Caledonia 20/1999) influenza virus in mice. Mannan is a non-toxic carbohydrate produced in most plants and yeasts, and it has been administered parentarally to over 200 humans as part of previous clinical trials in cancer, without adverse events [7-9]. Based on previous reports that IN immunisation of mice with mannan conjugated to antigen induces both mucosal IgA and serum IgG [10], mice were vaccinated via the intranasal route. Our initial studies focused on vaccination with preparations comprised of whole influenza virus conjugated to oxidised mannan, in an effort to target the virus directly to DCs, via the mannose receptor [11,12]. Surprisingly, while H1N1 conjugated to mannan was no more immunogenic than H1N1 alone, mixing free mannan with H1N1 yielded substantially greater serum IgG and IgA titres, and IgA titres in the lung.

After determining that extending the intervals between immunisations enhanced the induction of lung IgA, we then tested varying amounts of mannan mixed with a constant low dose of H1N1; while a small amount of mannan had proven unable to adjuvant a small amount of H1N1, we hypothesised that a large amount of mannan may be able to. Further, it was hypothesised that using the protracted immunisation schedule, substantial lung IgA and serum IgG may be induced after two immunisations, without the need for a third. These strategies proved successful, with two intranasal immunisations of 1 μg of H1N1 mixed with 100 μg of mannan inducing lung IgA, serum IgG_1_ and IgG_2a_, and serum immunity capable of neutralising the haemagglutination capacity of the virus *in vitro*.

## Methods

### Influenza virus and mice

Egg-grown H1N1 (A/New Caledonia/20/1999) virus purified by sucrose gradient, concentrated, and inactivated with ß-propiolactone was kindly provided by the WHO Collaborating Centre for Reference and Research on Influenza (North Melbourne, Australia). All mice were female BALB/c sourced from the Walter and Elisa Hall Institute (Melbourne, Australia), and were 8–10 weeks of age at first immunisation. All mouse-work was conducted at the Austin Hospital Animal Facility (Melbourne, Australia) in accordance with an animal ethics application approved by the Austin Animal Ethics Committee.

### H1N1 mannan conjugates and H1N1 mannan mixes

Whole inactivated H1N1 was conjugated to oxidised mannan via the conjugation method described in Stambas *et al*. [10]. Mannan (1 mL of 14 mg/mL) in 0.1 M phosphate pH 6.0 was oxidised with the addition of 0.1 M sodium periodate (100 μL in water) in the dark at 4°C for 1 hr. The mixture was quenched with 10 μL ethanediol and reacted for a further 30 min as before. The oxidised mannan mixture was passed through a PD10 column (GE Biosciences) pre-equilibrated with 0.05 M bicarbonate pH 9.0 to remove byproducts. The eluted 2 mL fraction of oxidised mannan (7 mg/mL) after void volume (2.5 mL) was collected. The resulting conjugate was used without further purification. In previous studies we have optimised the conjugation of mannan to recombinant cancer-associated antigens [13] as well as bacterial [10] and viral proteins. The periodate oxidation condition for mannan was chosen such that aldehyde residues are generated from only a fraction of oxidised mannose units of the mannan, without affecting its C-type lectin binding activity [14-17]. For a complex antigen such as a whole inactivated virus we expect the majority of conjugation of mannan aldehyde groups to take place only at the exposed amino groups, forming Schiff base linkages. To identify optimal conjugation conditions, various amounts of whole virus (36 μg, 18 μg and 9 μg) were separately reacted with 350 μg of oxidised mannan, and the resulting preparations were analysed by gel electrophoresis. The oxidised mannan-H1N1 conjugates were filter-sterilised and then aliquoted and stored at −20°C. The concentration of H1N1 was based on the total amount of H1N1 used for conjugation. Mixtures of H1N1 and mannan were generated by diluting the H1N1 stock (from 2.7 mg/mL) and the mannan stock (from 14 mg/mL) in sterile PBS, such that the desired dose of each was contained per 50 μL; the volume of each individual vaccination.

### Immunisation method

All immunisations were administered via the intranasal route. While completely anaesthetised (via methoxyfluorane inhalation) and held upright, 5-μL drops were gently pipetted alternately into each nostril. The total volume of every immunisation was standardised to 50 μL, regardless of H1N1 dose or mannan dose. All mannan used throughout the study was derived from *Sacharomyces cerevisiae*, and sourced from Sigma (product# M7504-5G).

### H1N1 mannan conjugate vs. mannan mixture

**Figure 1:**
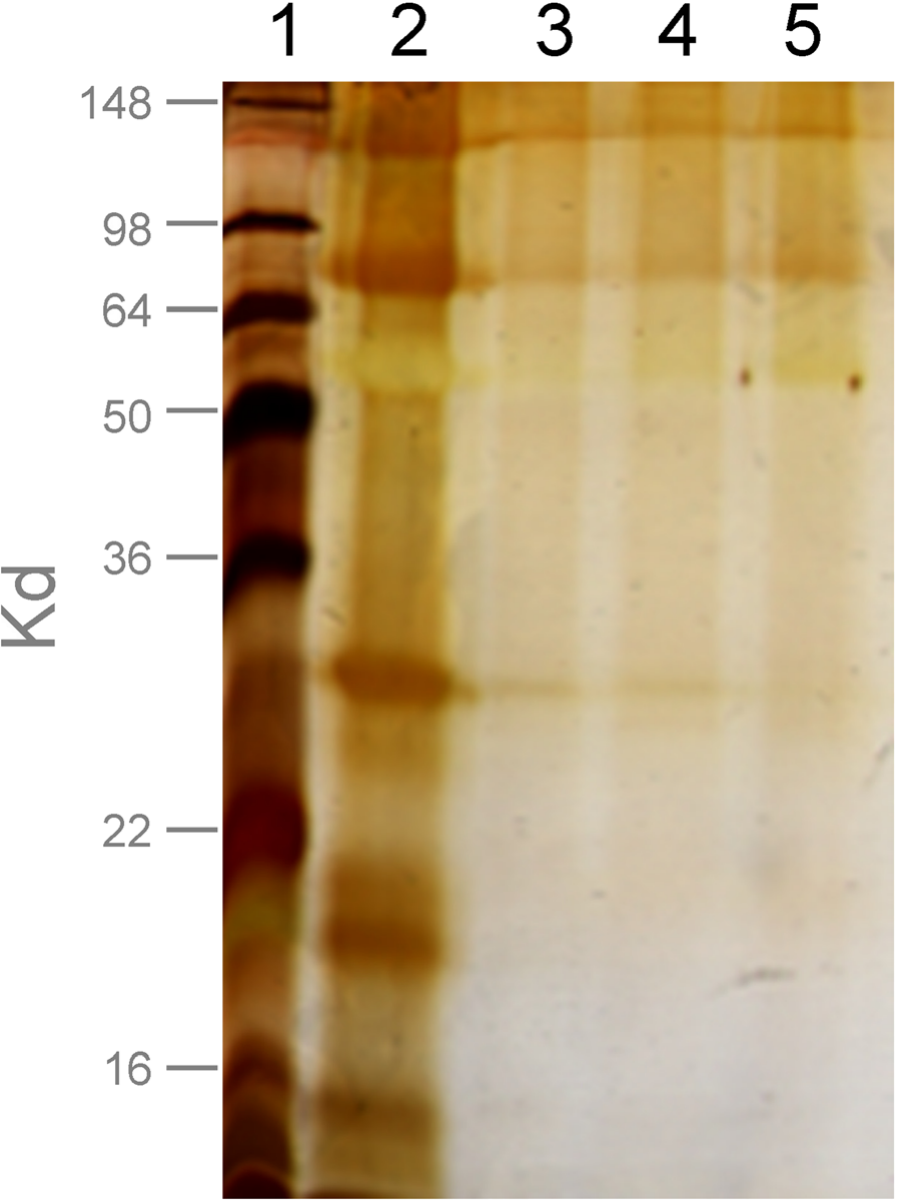
**Efficiency of oxidised mannan-H1N1 conjugation.** Conjugates of H1N1 and oxidised mannan (H1N1_OxMan) were generated at ratios of 1:10, 1:5 and 1:2.5 and analysed by gel electrophoresis followed by silver staining. Lane 1 = standards, 2 = H1N1 alone, 3 = 1:10, 4 = 1:5, 5 = 1:2.5.

To ascertain the immunogenicity of H1N1 conjugated to oxidised mannan or mixed with mannan with regard to induction of serum IgG, groups of 5 mice were immunised intranasally with H1N1 alone, H1N1 conjugated to mannan (H1N1_OxMan) or H1N1 mixed with mannan (H1N1 + mannan). Initially, two doses of virus were tested, 0.4 μg and 10.0 μg. When generating the H1N1_OxMan conjugates, a 10:1 ratio of mannan:H1N1 was used (see Figure 1). Thus, in the comparable ‘mannan mixed’ conditions this ratio was maintained; mice receiving 0.4 μg of H1N1 received 4 μg of mannan, and those receiving 10 μg of H1N1 received 100 μg of mannan. Mice were immunised twice, 10 days apart, then serum was collected 5 days after the second immunisation and assayed for anti-H1N1 IgG.

To investigate the potential for mannan to adjuvant the induction of IgA, groups of 9 mice were then immunised three times (days 0, 10 and 17) with 10 μg of H1N1 either alone, conjugated to 100 μg of mannan or mixed with 100 μg of mannan. Serum was harvested from all mice 7 days after the final immunisation, and lung washes were performed on 5 mice from each group. All samples were assayed for anti-H1N1 IgA by ELISA.

### Mannan mixture with extended immunisation intervals

We then assessed whether increasing the time between immunisations and delaying lung wash harvesting yielded greater respiratory tract IgA, as isotype conversion to IgA is known to occur over weeks, rather than days [18]. A subgroup of mice was immunised with 10 μg of H1N1 mixed with 100 μg of mannan three times at 14-day intervals, and lung washes were collected 14 days after the final immunisation.

### Increased mannan dose in H1N1 mixture

We then assessed whether a large amount of mannan could boost the immune response to a small amount of antigen. We also tested whether by using the previously investigated extended interval between immunisations (14 days), substantial serum IgG and lung IgA were induced in mice after two immunisations, negating the requirement for a third. Mice were immunised with 1 μg of H1N1 mixed with 10, 50 or 100 μg of mannan twice, 14 days apart. Fourteen days after the second immunisation, serum was harvested and assayed for anti-H1N1 IgG.

### Serum and bronchio-alveolar-lavage collection

Serum was collected via retro-orbital bleed as described in Donovan *et al.* [19]. Bronchio-alveolar-lavage (BAL) fluid was collected after mice were euthanised via an intraperitoneally-administered preparation consisting of 66 μL xylazil, 166 μL ketamine, and 266 μL saline. Tissue was removed to expose the upper trachea, and a small incision was made therein. With the aid if a blunt needle attached to a 1 mL syringe, 1 mL of PBS was gently flushed into the lungs, and drawn back out.

### ELISA determination of antibody titres

ELISAs were performed using the HRP/TMB system. Plates were coated with whole inactivated H1N1 (A/New Caledonia/20/1999) at a concentration of 1 μg/mL. Total anti-H1N1 IgG was detected using directly HRP-conjugated rat anti-mouse-IgG (GE healthcare, product # RPN1231V) and IgG_1_, IgG_2a_ and IgA were detected using biotin-labelled primary antibodies from Pharmingen (product numbers 553441, 553388 and 556978 respectively), and secondary streptavidin-HRP from GE healthcare (product #346480). End-titre was defined as the last value in the titration to remain above the corresponding control value, where the control was calculated as the mean OD values + 2SD of naive mouse serum samples (3–5 mice) at each titration point.

### Haemagglutination inhibition assays

HI assays were performed according to standard protocols [20]. Sera were pre-treated with receptor destroying enzyme (RDE) II (Deka Seiken Co. Ltd., Tokyo, Japan) at a ratio of 1:4 (v/v) at 37°C for 16 hrs, then the enzyme was inactivated by the addition of an equal volume of 54.4 mM tri-sodium citrate (Ajax Chemicals, Australia), and incubation at 56°C for 30 min. At room temperature, 25 μL of an A/New Caledonia/20/1999 virus preparation was added to 25 μL of the RDE-treated serum preparation, then this solution was titrated in two-fold dilutions in PBS from an initial serum:diluent ratio of 1:10 to a final ratio of 1:1280. Following a 1-hr incubation, 25 uL of a 1% (v/v) suspension of turkey RBCs was added to each well. Haemagglutination was assessed via standard methods [20], after 30 min. Where no neutralising antibodies were present RBC agglutination proceeded uninhibited, but where anti-haemagglutinin (HA) serum immunity had been generated, neutralising antibody bound to the HA protein, inhibiting its ability to agglutinate the RBCs. Titres were defined as the reciprocal of the highest dilution of serum where haemagglutination was prevented.

## Results

### H1N1/oxidised-mannan conjugates (H1N1_OxMan)

Before the administration of mannan conjugates to mice, the most effective ratio of oxidised mannan:H1N1 with regard to conjugation efficiency was determined, as described in the *Methods* section, above. The ratio of 39 μg of inactivated H1N1 to 350 μg of oxidised mannan (Figure 1, lane 3) resulted in the most mannosylation as judged by the replacement of discrete viral protein bands with a smear, due to glycosylation with oxidised mannan, as described in Apostolopoulos *et al.* [13] (Figure 1).

### Immunogenicity of H1N1_OxMan conjugates and H1N1 + mannan mixes in mice

**Figure 2:**
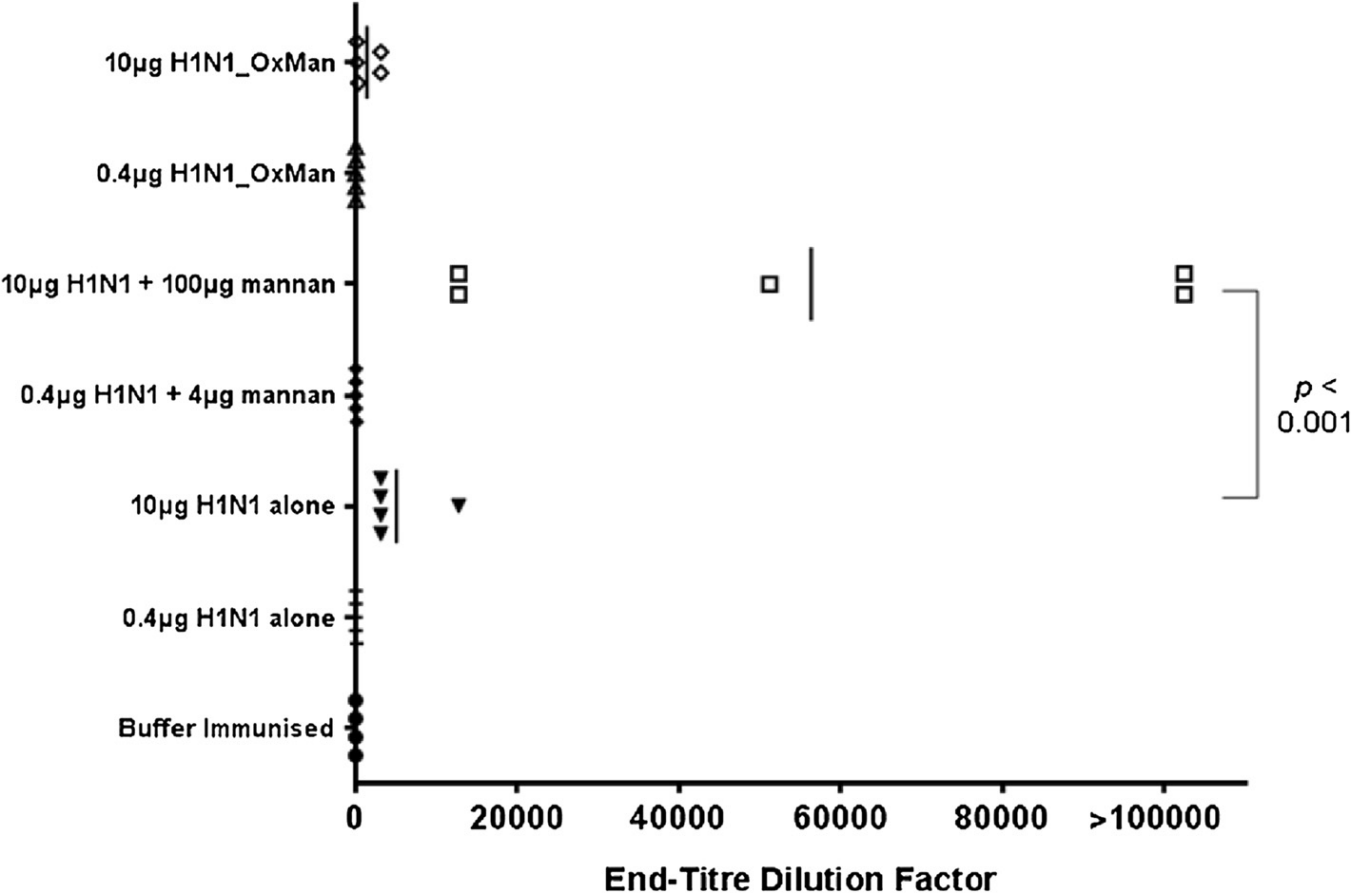
**Immunogenicity of H1N1, H1N1_OxMan and H1N1 + mannan.** Mice were immunised intranasally on days 0 and 10 with 0.4 or 10.0 μg of H1N1 alone, H1N1_OxMan or H1N1 mixed with mannan (H1N1 + mannan). Serum was harvested on day 15 and anti-H1N1 IgG activity was determined by ELISA.

Unexpectedly, in the initial experiment H1N1_OxMan was not more immunogenic than H1N1 alone with regard to serum IgG, however 10 μg of H1N1 mixed with 100 μg of mannan induced higher titres of serum IgG than 10 μg of H1N1 alone (*p* < 0.01, Bonferroni-corrected multiple comparisons test) (Figure 2). No responses were detected in any of the mice immunised with 0.4 μg H1N1 alone, conjugated to 4 μg mannan, or mixed with 4 μg mannan. While intranasal immunisation with mannan alone was not included in this experimental series, we have previously ascertained that this induces no H1N1-specific antibody as determined by ELISA and HI assays (data not shown).

### Mucosal antibody responses to H1N1_OxMan conjugates and H1N1+ mannan mixes

**Figure 3:**
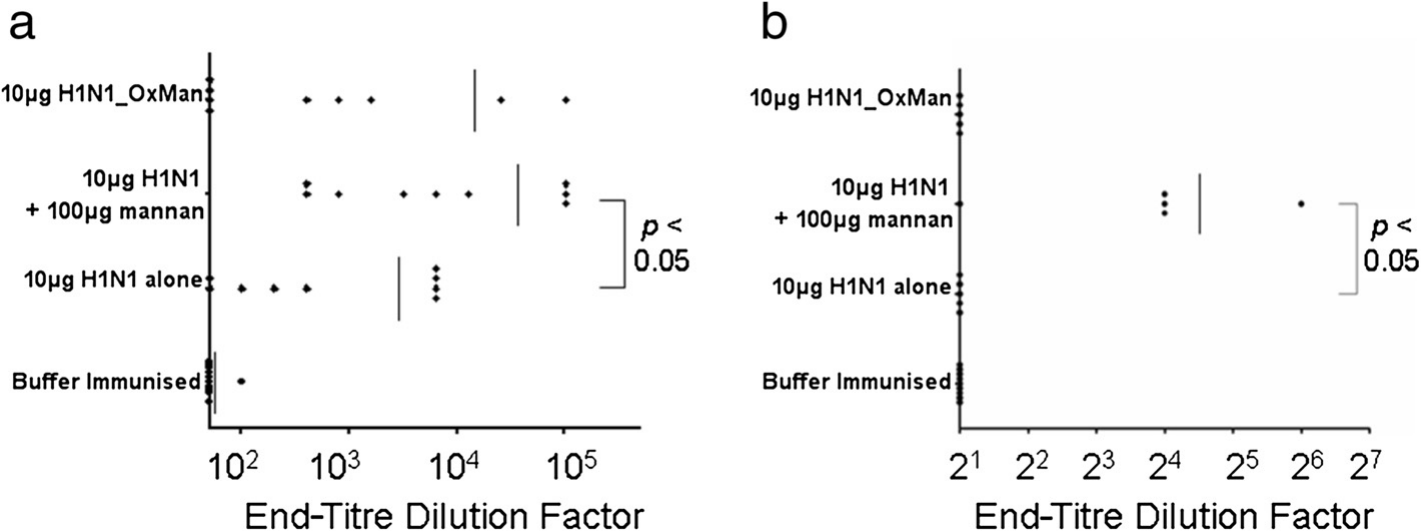
**Mucosal antibody responses to H1N1_OxMan and H1N1 + mannan.** Mice were immunised intranasally on days 0, 10 and 17 with 10 μg of either H1N1 alone, H1N1_OxMan or H1N1 + 100 μg mannan. Ten days after the final immunisation, **(a)** serum samples and **(b)** lung-wash samples were harvested and tested for anti-H1N1 IgA activity by ELISA assay.

After immunising groups of 9 mice three times (at days 0, 10 and 17) with 10 μg of H1N1 either alone, conjugated to 100 μg of mannan or mixed with 100 μg of mannan, serum was harvested from all mice 7 days after the final immunisation, and lung washes were performed on 5 mice from each group. While immunisation with H1N1 alone and H1N1_OxMan induced low levels of serum IgA in some mice, H1N1 mixed with mannan induced serum IgA in all mice (Figure 3a). Mucosal IgA was only detected in the lungs of mice immunised with H1N1 mixed with mannan (Figure 3b).

### Optimisation of the immunogenicity of H1N1 + mannan mixtures

**Figure 4:**
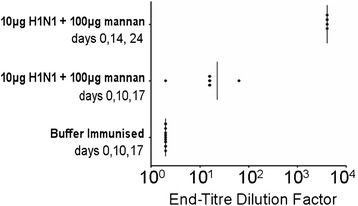
**Differential vaccination kinetics and mucosal IgA induction.** Mice were immunised intranasally with 10 μg of H1N1 mixed with 100 μg of mannan either on days 0, 10 and 17 with lung washes collected on day 22, or on days 0, 14 and 28 with lung washes collected on day 32. The amounts of anti-H1N1 IgA in the lung washes of individual mice were determined by ELISA.

A subgroup of mice was immunised with 10 μg of H1N1 mixed with 100 μg of mannan three times at 14-day intervals, and lung washes were collected 14 days after the final immunisation. Increasing the intervals between immunisations and sample harvesting to 14 days resulted in higher lung IgA titres (Figure 4), and was adopted thereafter, throughout the rest of the study.

**Figure 5:**
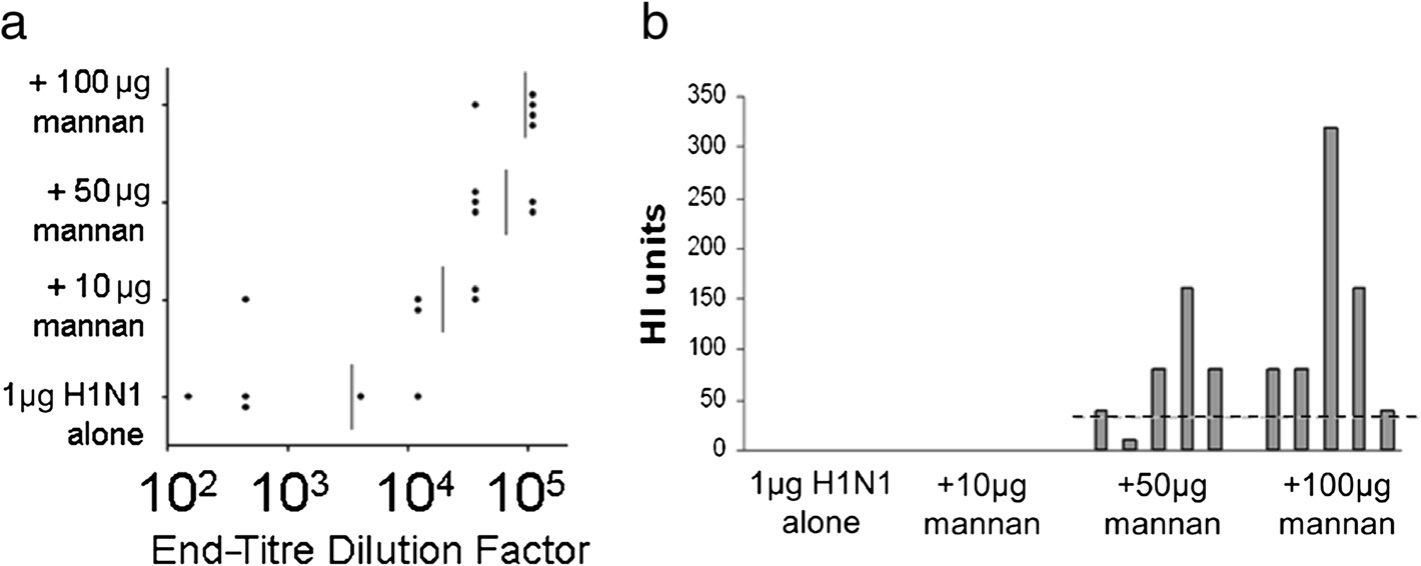
**Optimisation of H1N1:mannan ratio and serum haemagglutination inhibition activity.** Groups of mice were immunised twice 14 days apart with 1 μg H1N1 mixed with various amounts of mannan, and 14 days after the last immunisation mouse sera were tested for **(a)** serum IgG antibody, and **(b)** haemagglutination inhibition activity.

While a small amount of mannan was unable to adjuvant a small amount of H1N1 (Figure 2), we hypothesised that a large amount of mannan may be able to boost the immune response to a small amount of antigen. Further, it was hypothesised that by delaying immunisation for 2-week intervals, substantial serum IgG may be evident in mice after two immunisations, without the requirement for a third. To test these hypotheses, mice were immunised with 1 μg of H1N1 mixed with either 10, 50 or 100 μg of mannan twice, 14 days apart. Fourteen days after the final immunisation, serum was harvested and assayed for anti-H1N1 IgG. One hundred micrograms of mannan was able to significantly adjuvant 1 μg of H1N1 after two immunisations (Figure 5a), and thereafter 100 μg of mannan was adopted as the standard dose, throughout the rest of the study.

To confirm that the antibody induced by mannan-adjuvanted H1N1 was likely to equate with *in vivo* protection, serum from the immunised mice was tested in an HI assay. Only serum from mice immunised with H1N1 adjuvanted with 50 or 100 μg of mannan was able to inhibit the ability of H1N1 to agglutinate turkey RBCs. In previous studies [21], a titre of ≥ 40 (indicated by the dashed line) correlated with 50% protection against experimental infection with partially attenuated challenge strains. In this study, no mice immunised with 1 μg H1N1 alone yielded HI titres of ≥ 40, while all mice immunised with 1 μg H1N1 + 100 μg of mannan yielded HI titres of ≥ 40 (*p* < 0.01, Fisher's exact test) (Figure 5b).

**Figure 6:**
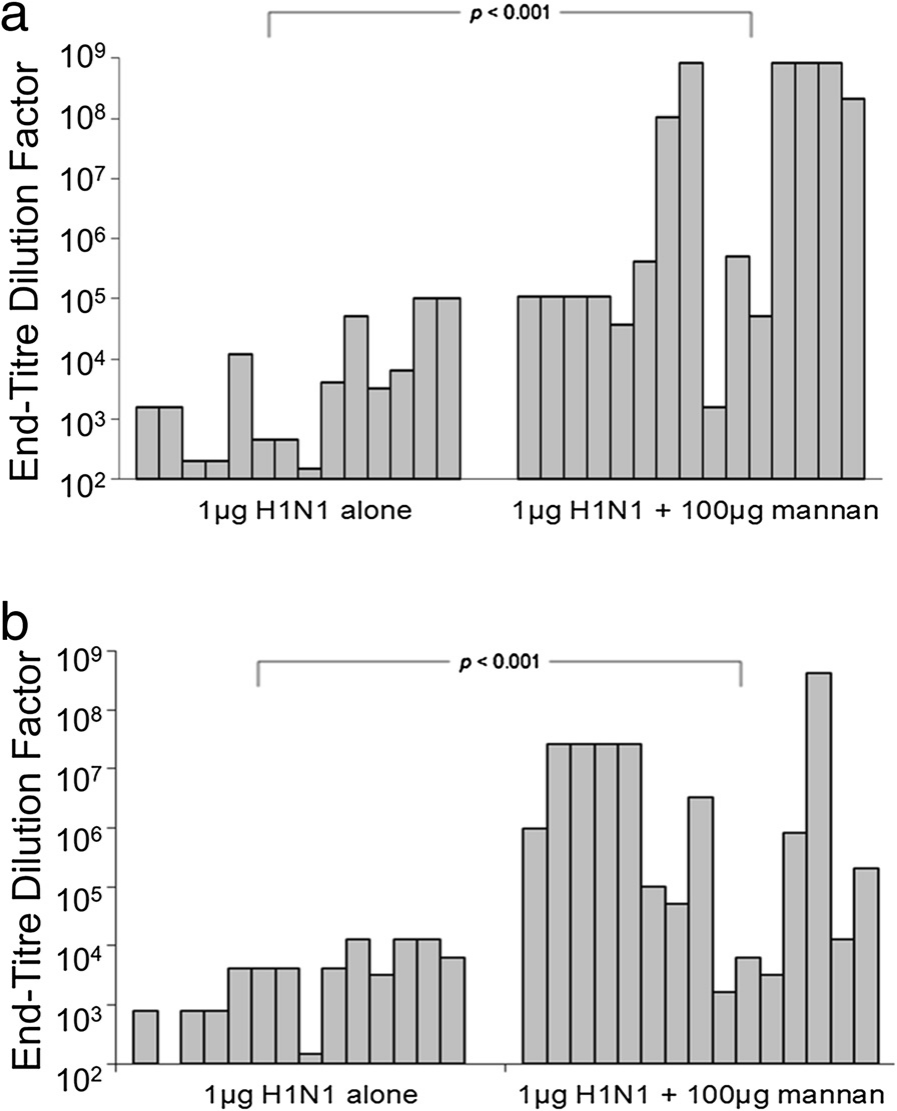
**IgG sub-class induction after immunisation with 1 μg of H1N1 adjuvanted with 100 μg of mannan.** Groups of mice were immunised twice 14 days apart with 1 μg of H1N1 alone, or mixed with 100 μg mannan, and 14 days after the last immunisation serum was harvested and assayed for anti-H1N1 **(a)** IgG_1_ and **(b)** IgG_2a_ by ELISA.

To statistically validate the hypothesis that 100 μg of mannan adjuvants antibody responses to 1 μg H1N1, larger groups of mice were immunised twice at 14-day intervals and serum was harvested 14 days after the second immunisation. IgG_1_ and IgG_2a_ isotypes were detected separately, and the titres of both isotypes were significantly enhanced via the inclusion of 100 μg of mannan (*p* < 0.001, Mann–Whitney test) (Figure 6).

**Figure 7:**
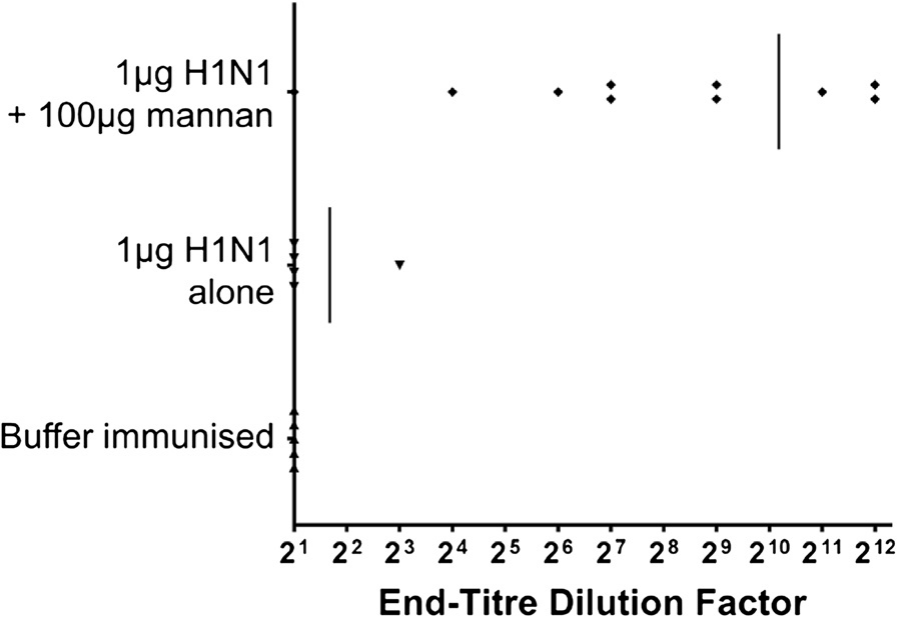
**Anti-H1N1 IgA in lung washes 14 days after two intranasal immunisations with 1 μg of H1N1 mixed with 100 μg of mannan.** Mice were immunised twice 14 days apart with 1 μg of H1N1 alone or mixed with 100 μg mannan, and 14 days after the last immunisation lung washes were harvested and assayed for anti-H1N1 IgA.

Lung washes were performed on a randomly selected subgroup of mice from the above experimental series, and assayed for IgA. IgA was evident in all but one of the mice immunised with H1N1 + mannan (*n* = 10), but was essentially absent in mice immunised with H1N1 alone (*n* = 5) (*p* < 0.01, Mann–Whitney test) (Figure 7). The high variability in lung IgA titres within the ‘+ mannan’ group suggests that 1 μg H1N1 may correspond to the approximate minimum H1N1 dose that can be expected to induce detectable lung IgA in the majority of mice under the conditions tested.

**Figure 8:**
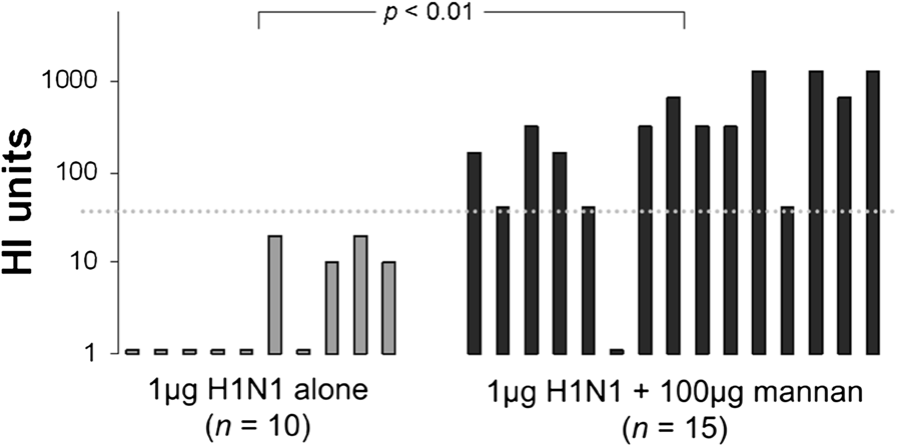
**Haemagglutination inhibition by serum from mice immunisation with 1 μg of H1N1 adjuvanted with 100 μg of mannan.** Groups of mice were immunised twice 14 days apart with 1 μg of H1N1 alone or mixed with 100 μg mannan, and 14 days after the second immunisation serum was harvested and assayed for its ability to inhibit the agglutination of turkey RBCs by H1N1 *in vitro*.

Serum samples from three separate trials in which mice were immunised with 1 μg of H1N1 alone or with 100 μg mannan intranasally 14 days apart were tested for their ability to inhibit HA-protein mediated haemagglutination. While none of the serum samples from mice immunised with H1N1 alone (*n* = 10) elicited HI titres of ≥ 40 (mean titre = 6), 93% of mice immunised with H1N1 + mannan mix (*n* = 15) elicited HI titres of ≥ 40 (mean titre = 456) (Figure 8).

## Discussion

When the threat of an H5N1 pandemic emerged in 2005, the World Health Organisation (WHO) indicated that research investigating flu adjuvants was a high priority. Identifying an effective ‘dose-sparing’ adjuvant was deemed important due to the potential emergence of new strains (reviewed in Fisman *et al*. [22]) and the lag time involved in producing vaccine in live embryonated chicken eggs.

In this study, the dose sparing potential of mannan as an adjuvant for influenza was explored. The initial strategy of conjugating mannan to whole influenza virus was investigated based on previous observations suggesting that this may promote targeting of antigen to DCs via the mannose receptor [11,12]. However, while mannan-conjugated whole virus proved no more immunogenic that virus alone, unexpectedly, ‘free mannan’ adjuvanted whole virus significantly. As our initial studies were focused on mannan-influenza conjugates, the ratio of mannan to H1N1 included in the ‘control’ (mannan mixed) conditions was kept proportional to the amount used to derive the conjugates; mice receiving low doses of H1N1 received proportionally low amounts of mannan. However, once it was ascertained that mannan functioned as an adjuvant when mixed with (rather than conjugated to) whole influenza virus, we abandoned the conjugation-based mannan to antigen ratio, and confirmed that 100 μg of free mannan was able to adjuvant a single microgram of whole inactivated H1N1.

We investigated intranasal influenza vaccination as this has been shown to stimulate the mucosal immune response and generate secretory IgA at the site of infection [23]. Encouragingly, in a large-scale multi-centre human trial comparing intranasal (FluMist®) and intramuscular (Sanofi Pasteur) immunisation against influenza infection, IN immunisation was more protective than intramuscular (IM) immunisation against naturally acquired infection in infants [24]. Notably, the intranasal route is also more amenable to ‘self-administration’ (via a nasal spray) than an intramuscular injection, which may prove advantageous during a pandemic caused by a rapidly spreading newly emergent influenza strain. Should a ‘booster’ (*i.e.* a second vaccination) be required for example, this could be supplied to the vaccinee at the time of their first vaccination (or subsequently by a pharmacist), to be self-administered 2 weeks after the first vaccine. Under this scenario, the first vaccine could also conceivably actually be self-administered under the careful guidance of a health-care professional, to ensure that the vaccinee was confident and capable of self-administering any subsequently required boosters. In a ‘similar vein’, in remote or disadvantaged communities where infrastructure and health professionals are scarce, a vaccine that negates the use of needles and could be self-administered would conceivably be advantageous.

While it has previously been shown that IN administration of mannan-conjugated proteins generates antigen-specific IgA in the lung [10], this is the first demonstration of this using whole virus as antigen and free mannan as adjuvant. The contribution of H1N1-specific lung IgA to protection against a live influenza challenge is currently under investigation. Both IgG_2a_ and IgG_1_ were induced by H1N1 + mannan in the present study, suggesting the induction of cellular as well as humoral immunity. This bodes well for the vaccine strategy, as it is widely held that the generation of effective ‘heterotypic’ (cross-strain) immunity will require induction of a functional T cell response. We are currently characterising the cellular immunity induced by immunisation with mannan + H1N1 via a series of short peptides derived from each of the core viral proteins.

To date we have been unable to definitively confirm that the mannose receptor is involved in the mechanism of adjuvantation by free mannan observed in the current study, and notably other innate receptors may also be involved [25]. Nevertheless, the observation that conjugation is not required in the context of influenza adjuvantation is fortuitous with regard to regulatory and production considerations; it would conceivably be much easier to achieve compositional standardisation across batches of GLP-produced vaccine if ‘production’ merely entailed mixing two homogenous GLP products together. Further, mannan is an adjuvant well suited to *en-masse* vaccination as it is very cheap, non-proprietary, easy to sterilise, store and transport, and has proven to be non-toxic to humans when delivered parenterally in clinical trials collectively comprising > 200 patients [7-9].

The recent emergence of ‘swine flu’ (H1N1s) has reinforced the need for a dose-sparing influenza adjuvant. In Australia [26] and New Zealand [27], after the rate of the initial wave of H1N1s infections peaked (in approximately June/early July 2009) it then declined, but the overall incidence of new infections remained significant, to the extent that H1N1s actually ‘displaced’ the seasonal A(H1N1) strain that had previously been predicted would account for the majority of infections. Despite the initial characterisation and isolation of the H1N1s strain occurring in April 2009 [28], ‘growing up’ enough effective doses of the vaccine was by necessity time-consuming, which contributed to the fact that vaccine did not become available to the general public until almost 6 months later, in early October. Before it became clear that H1N1s posed no greater risk of mortality than historical variants of the H1N1 ‘human’ flu, the British Government evidently intended to immunise more than half of its entire population against H1N1s [29]. This would have been a massive endeavour, the efficacy of which would have depended largely on the speed at which it could have been implemented, which would in turn have been limited by the rate of production of effective vaccine doses. Implementation of a dose-sparing influenza adjuvant that allowed, for example, a five to ten-fold reduction in the amount of virus constituting a protective dose would significantly reduce the time between isolating a potentially pandemic influenza strain, and generating enough virus to immunise the population *en-masse*.

## Conclusions

Mannan successfully adjuvanted H1N1 vaccination in mice, rendering otherwise weakly immunogenic doses inductive of strong serum IgG, and IgA in the lung. As mannan is cheap, non-toxic, non-proprietary, abundant and easy to transport, it represents an attractive candidate adjuvant for rendering small doses of human influenza vaccines more immunogenic (‘dose sparing’) in the event of a pandemic involving a highly virulent and/or transmissible strain; the results of the current study in mice suggest that further studies in larger animals and/or humans are warranted.
